# The Auxiliary Diagnostic Value of a Novel Wearable Electrocardiogram-Recording System for Arrhythmia Detection: Diagnostic Trial

**DOI:** 10.3389/fmed.2021.685999

**Published:** 2021-06-24

**Authors:** Shaomin Zhang, Hong Xian, Yi Chen, Yue Liao, Nan Zhang, Xinyu Guo, Ming Yang, Jinhui Wu

**Affiliations:** The Center of Gerontology and Geriatrics, West China Hospital, Sichuan University, Chengdu, China

**Keywords:** arrhythmia, atrial fibrillation, AMAZFIT, detection, diagnostic trial

## Abstract

**Background:** AMAZFIT^®^, a novel wearable electrocardiogram (ECG)-recording system is used for the measurement, acquisition, and storage of single-lead cardiac waveforms for adults. The aim of the study was to evaluate the accuracy of AMAZFIT^®^ for diagnosing arrhythmia in older patients.

**Methods:** From May to December 2019, we recruited 291 elderly individuals with an average age of 78±10 years old, and 41.9% women. All cardiac waveforms were obtained from the AMAZFIT^®^ which included limb and chest leads. Two trained technicians reviewed all ECG data to determine cardiac rhythm using standard diagnostic criteria. We evaluated the accuracy of AMAZFIT^®^ for identifying arrhythmia by comparing the sensitivity, specificity, accuracy, positive predictive value (PPV), negative predictive value (NPV), and positive and negative likelihood ratios with those of a standard 12-lead ECG.

**Results:** Of the 291 participants, 197 older adults had arrhythmias, including AF (*n* = 119), first-degree AVB (*n* = 28), PACs (*n* = 25), and PVCs (*n* = 28). Three of these participants had arrhythmias of AF and PVCs. Chest lead data from 100% and limb lead data from 4.7% of the participants were analyzed. An evaluation of AMAZFIT^®^ for atrial fibrillation (AF) reported a sensitivity, specificity, PPV, NPV PLR, and negative likelihood ratio (NLR) of 93.28, 95.35, 93.28, 95.35, 20.06, and 0.07%, respectively. AMAZFIT^®^ also demonstrated excellent sensitivity for premature atrial contractions (PACs) (84.00%) and premature ventricular contractions (PVCs) (89.29%). However, the device demonstrated a low sensitivity for first-degree atrioventricular block (32.14%).

**Conclusions:** The AMAZFIT^®^ showed significantly higher sensitivity and specificity for AF, PACs, and PVCs. This portable ECG-recording device based on an algorithm has a potential auxiliary diagnostic value for identifying arrhythmia compared with a standard 12-lead ECG device.

## Introduction

The incidence and prevalence rates of arrhythmias, which include atrial fibrillation (AF), premature heart beats, and conduction block, are showing increasing trends, particularly in the elderly population. AF is one of the world's most common serious cardiac rhythm problems. Arrhythmias are significantly associated with increased morbidity and higher mortality (1–3). The prevalence of arrhythmia is doubled in people over 40 years of age (4, 5). Earlier identification of AF may reduce the risk of stroke and other consequences. A diagnostic challenge is that AF is often asymptomatic (6). An invasive cardiac monitoring strategy is the gold-standard for high-risk patients (7, 8). Insertable leadless cardiac monitors are associated with higher AF detection rates; however, this strategy is not easily adopted due to its inconvenience, and invasive characteristics, among other reasons.

Wearable arrhythmia detection devices are becoming increasingly popular (9). There is an increasing interest in the use of smartwatch-based technology for AF detection, with some initial data indicating that these devices may be used for population-level AF screening. While there is high clinician interest in the diagnostic precision from these devices, there is a paucity of data that evaluates their accuracy for the diagnosis of arrhythmia (10).

Some smartwatches such as the Apple Watch, Kadia Band, Kadia Mobile, and Simband, have been used for cardiac rhythm analyses (11–14). Unlike these smartwatches, the AMAZFIT^®^ (Huami Corporation, Anhui, China) can collect single-lead cardiac waveforms from the chest lead, which could be used for the measurement, acquisition, and storage of single-lead cardiac waveforms for adults. We aimed to evaluate the accuracy of the AMAZFIT^®^ as a detection tool for the diagnosis of arrhythmia in older patients.

## Methods

This was a diagnostic trial. A 12-lead ECG was used as the gold-standard to evaluate the diagnostic value of a wearable dynamic ECG recorder (AMAZFIT^®^) for the detection of arrhythmia.

### Study Population

We continuously invited patients (≥ 65 years of age) who were admitted to the Center of Gerontology and Geriatrics of West China Hospital from May to December 2019 who had a complaint of palpitation to participate in this study. The exclusion criteria were as follows: (1) a history of cognitive impairment (MMSE < 10 score); (2) a history of skin disorders around the wrist or chest; (3) those who had unstable vital signs; and (4) those who refused to participate. All patients gave written informed consent.

### Data Collection

For all participants, after obtaining informed consent, baseline demographic, clinical comorbidities and medications were abstracted from their medical records by a trained staff member. The arrhythmia and the type of arrhythmia were determined according to the results of conventional 12-lead ECG.

**Figure 1:**
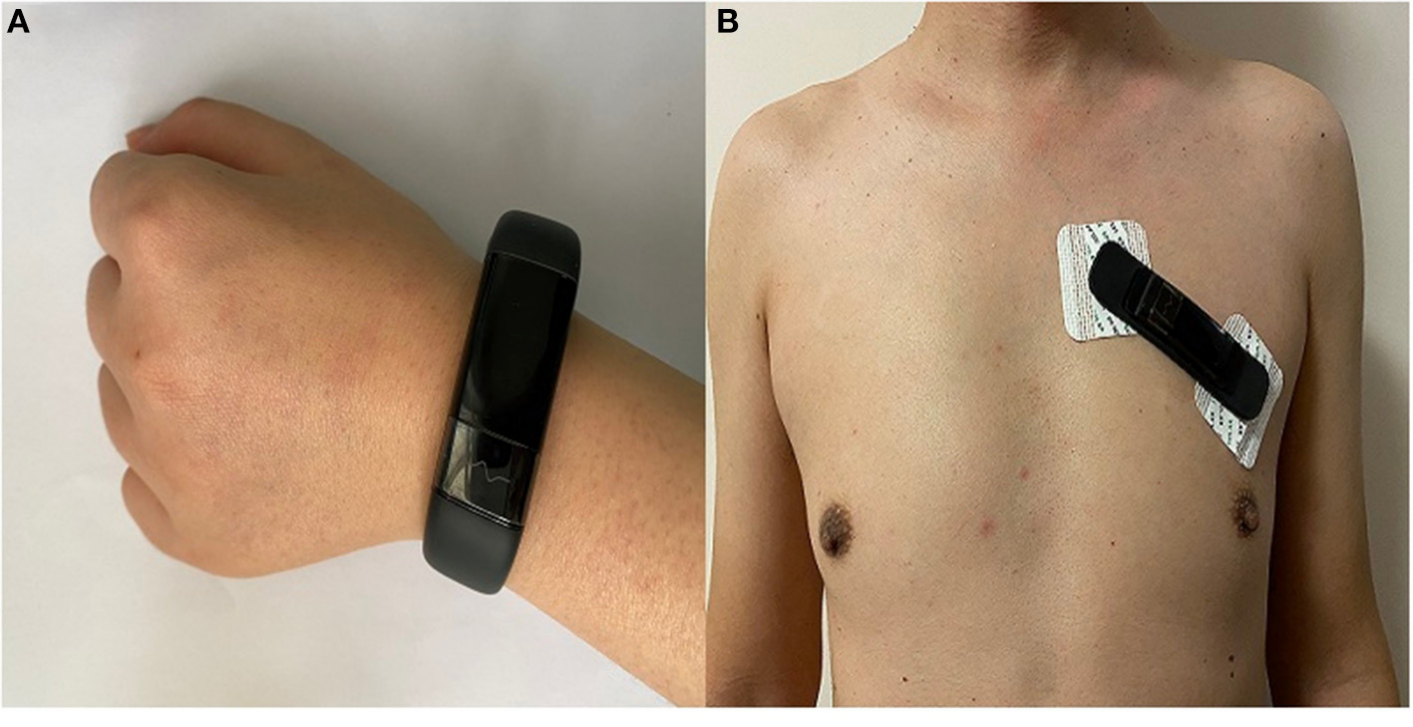
AMAZFIT^®^ location diagram. **(A)**: wrist **(B)**: chest wall.

All participants were asked to hold the AMAZFIT^®^ wearable dynamic ECG recorder (Model No DL4310/03; Production Permit No 20180012; Product Registration No 20182210012) on their wrist and chest wall for 1 min, during which time the pulse waveform was recorded in the AMAZFIT^®^ Health App. Participants wore an AMAZFIT^®^ on either wrist to collect limb lead data, which was firmly secured above the ulnar styloid. To collect chest lead data, the AMAZFIT^®^ was applied with medical electrode stickers. AMAZFIT^®^ is attached to the left side of the chest wall as shown in Figure 1. The mobile phone software was connected to the AMAZFIT^®^, and then the chest patch mode was selected to start the measurement.

**Figure 2:**
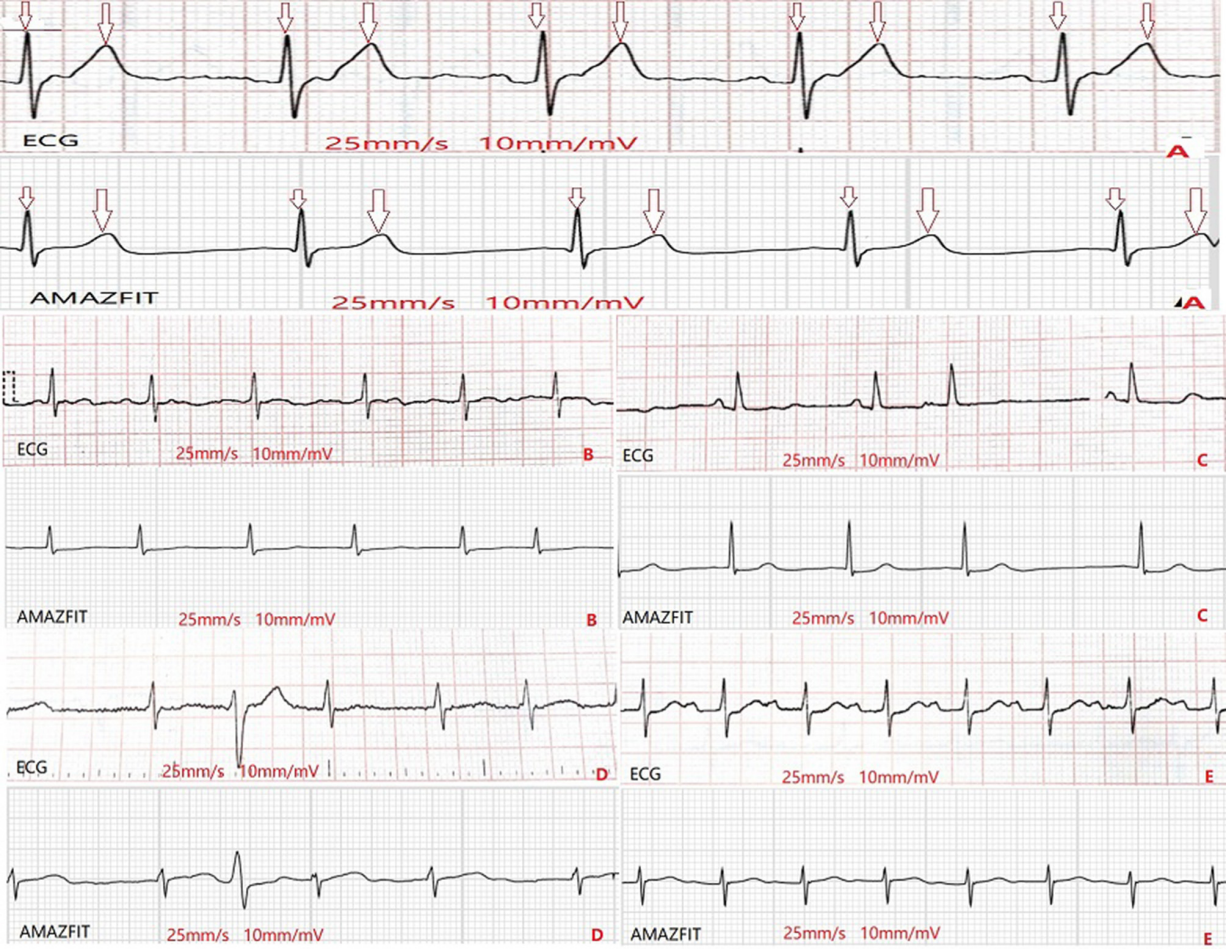
Comparison of the waveforms from ECG and from AMAZFIT^®^**. (A)**: sinus rhythm; **(B)**: AF; **(C)**: PACs; **(D)**: PVCs; **(E)**: first-degree AVB. AF, arterial fibrillation; PACs, premature arterial contractions; PVCs, premature ventricular contractions; AVB, atrioventricular block.

At the same time, all participants were simultaneously acquiring regular 12-lead ECG waveforms. The 12-lead electrode has 10 electrode heads, which are placed on the human chest C1, C2, C3, C4, C5, C6, and the limbs RA(right arm), LA(left arm), RL(right leg), LL(left leg) positions, thereby obtaining 12-lead ECG(I, II, III, aVR, aVL, aVF, V1, V2, V3, V4, V5, and V6). For both techniques, the high pass and low pass filters used for ECG signal processing were respectively 0.6 and 25 Hz for 12-lead ECG and 0.67 and 40 Hz for the AMAZFIT^®^. Two trained ECG technicians reviewed all ECG data that was collected by AMAZFIT^®^ to determine heart rhythm using standard criteria. In case of disagreement about the diagnosis between the reviewers, an electrophysiologist was consulted. The comparison between conventional 12-lead ECG and chest lead ECG data collected by AMAZFIT^®^ are shown in Figure 2.

### Statistical Analysis

Statistical analyses were performed using the SPSS 22.0 software program. Continuous variables were described as means ± standard deviations and categorical variables were described as numbers (percentages). We then calculated test characteristics, including sensitivity, specificity, accuracy, PPV, NPV, positive likelihood ratio and negative likelihood ratio for the AMAZFIT^®^ wearable dynamic ECG recorder detection for AF, first-degree AVB, PACs, and PVCs compared with the expert reviewer diagnosis (criterion standard) of sinus rhythm based on 12-lead ECG.

## Results

The characteristics of the 291 participants included in our study are shown in Table 1. The mean age of the cohort was 78 ± 10 years, and 41.9% were women. A total of 119 participants were included in the AF cohort. There was no atrial flutter cases in the AF cohort. The other arrhythmia cohort included 81 participants with: first-degree atrioventricular block (*n* = 28), PACs (*n* = 25), and PVCs (*n* = 28), while the normal ECG cohort included 94 participants.

**Table 1:** Baseline characteristics of the study population.

	**AF**	**AVB**	**PACs**	**PVCs**	**Normal ECG**
	**(*n* = 119)**	**(*n* = 28)**	**(*n* = 25)**	**(*n* = 28)**	**(*n* = 94)**
Age, mean (SD), years	76 ± 10	84 ± 9	80 ± 10	76 ± 9	79 ± 9
Female	56(47.1)	7(25.0)	13(52.0)	6(21.4)	38(40.4)
**Comorbidity**					
Hypertension	65(54.6)	23(82.1)	19(76.0)	20(71.4)	59(62.8)
COPD	22(18.5)	11(39.3)	4(16.0)	5(17.9)	24(25.5)
Diabetes	19(16.0)	12(42.9)	5(20.0)	5(17.9)	25(26.6)
Coronary artery disease	27(22.7)	12(42.9)	7(28.0)	6(21.4)	26(27.7)
Stroke	22(18.5)	6(21.4)	8(32.0)	6(21.4)	9(9.6)
**Anti-arrhythmic drug**					
β- blocker	45(37.8)	7(25.0)	11(44.0)	14(50.0)	28(29.8)
CCB	34(28.6)	13(46.4)	13(52.0)	10(35.70)	24(25.5)
Digoxin	18(15.1)	0(0.0)	0(0.0)	2(7.1)	1(1.1)
Amiodarone	21(17.6)	1(3.6)	6(24.0)	3(10.7)	7(7.5)

*AF, atrial fibrillation; AVB, atrioventricular block; PACs, premature atrial contraction; PVCs, premature ventricular contraction; ECG, electrocardiography; COPD, chronic obstructive pulmonary disease; CCB, calcium channel blockers*.

**Figure 3:**
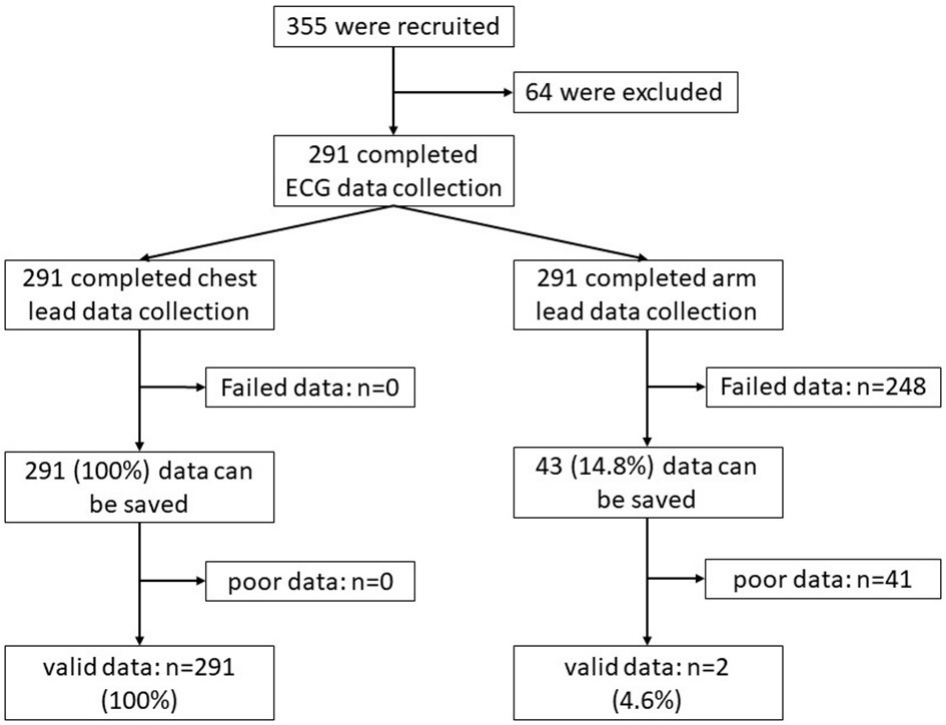
Participant selection and summary of ECG results.

We used AMAZFIT^®^ to collect cardiac waveform data for all participants, including limb lead and chest lead data. Chest lead data were successfully collected from 291 participants, while limb lead data were successfully collected from 43 participants. A total of 291 (100%) chest lead data recordings were analyzed, while only two (4.7%) limb lead data recordings were analyzed (Figure 3).

Compared with standard 12-lead ECG recordings, the AMAZFIT^®^ detected AF, first-degree AVB, PACs, PVCs and normal ECG with similar accuracy (Table 2). An evaluation of the wearable dynamic ECG recorder for AF reported a sensitivity of 93.28%, a specificity of 95.35%, a PPV of 93.28%, an NPV of 95.35%, a PLR 20.06 and an NLR 0.07. This indicates that the smartwatch has excellent sensitivity and specificity for the detection of an irregular pulse from AF. The smartwatch also demonstrated superior sensitivity for PACs (84.00%) and PVCs (89.29%), respectively. However, the device demonstrated a low sensitivity for first-degree AVB (32.14%). The specificity of the AMAZFIT for first-degree AVB (97.72%), PACs (96.62%), PVCs (93.92%), and normal ECG (89.34%) detection was excellent.

**Table 2:** Diagnostic accuracy of AMAZFIT^®^ against ECG.

	***n***	**Sensitivity**	**Specificity**	**Accuracy**	**PPV**	**NPV**	**PLR**	**NLR**
		**(100%)**	**(100%)**	**(100%)**	**(100%)**	**(100%)**		
Arrhythmia	197	89.34	92.55	97.59	96.17	80.56	11.99	0.12
AF	119	93.28	95.35	94.50	93.28	95.35	20.06	0.07
First-degree AVB	28	32.14	97.72	91.41	60.00	93.12	14.10	0.69
PACs	25	84.00	96.62	95.53	70.00	98.47	24.00	0.16
PVCs	28	89.29	93.92	93.47	60.98	98.80	14.69	0.11
Normal ECG	94	92.55	89.34	90.38	80.56	96.17	8.68	0.08

*PPV, positive predictive value; NPV, negative predictive value; PLR, positive likelihood ratio; NLR, negative likelihood ratio; AF, atrial fibrillation; AVB, atrioventricular block; PACs, premature atrial contraction; PVCs, premature ventricular contraction; ECG, electrocardiography*.

**Figure 4:**
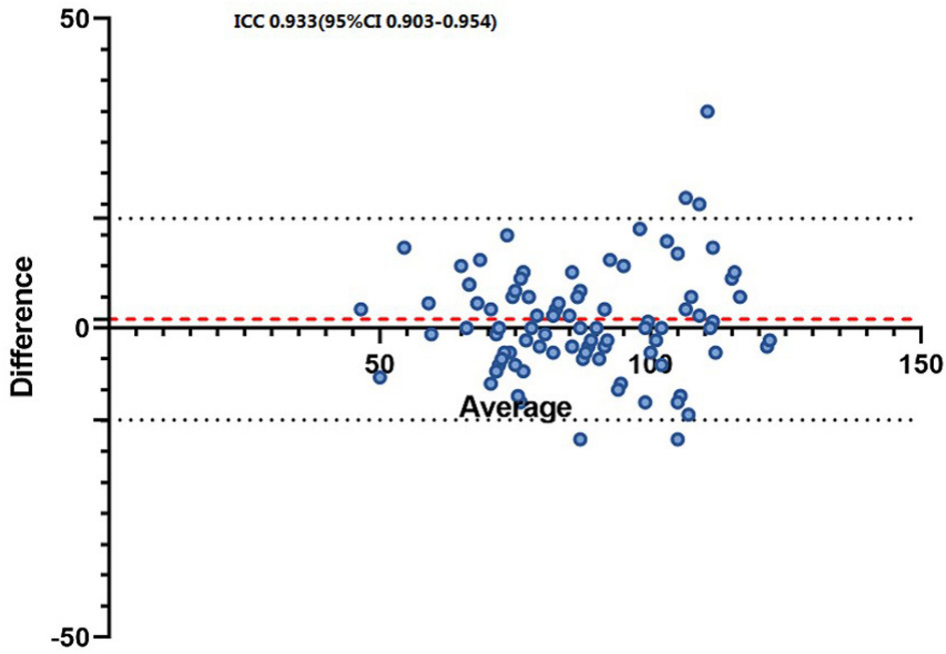
Bland-Altman plots of the heart ratio between the ECG and AMAZFIT^®^.

We measured the HR of patients with AF using AMAZFIT^®^ and 12-lead ECG. The intraclass correlation coefficients (ICC) of each device with a Bland-Altman plot of the induced SVT HR measurements are shown in Figure 4.

## Discussion

Our findings revealed that detection of arrhythmia with AMAZFIT^®^ was feasible, and showed high diagnostic accuracy. The AMAZFIT^®^ smart watch displayed excellent sensitivity for AF, PACs and PVCs, and a low sensitivity for first-degree AVB. In addition, we measured the HR of patients with AF using AMAZFIT^®^ and with a 12-lead ECG. The results showed that the device can accurately measure the HR of AF similarly to the 12-lead ECG.

Arrhythmia is a common disease in the elderly, and AF is one of the world's most common serious heart rhythm problems. AF increases the risk of stroke and heart failure in elderly patients, which in turn reduces the quality of life and increases the burden of care for elderly patients (15, 16). Although the 12-lead ECG remains the gold-standard diagnostic test for AF, a major challenge in diagnosis is the often paroxysmal nature of arrhythmia, particularly in its early stages. As such, periodic 24-h Holter monitoring is frequently used to determine the adequacy of rate control and guides medical therapy. The most commonly prescribed non-invasive AF monitors, including Holter and 30-day event monitors, have severe methodological shortcomings that result in low compliance, diagnosis delay, high cost, and adverse patient outcomes. Monitors with automated AF detection capabilities are approved for clinical use, but the invasive nature, cost, and technical limitations have limited their widespread application (17, 18).

There is increasing interest in the use of smartphone-based technology in arrhythmias especially in AF detection with numerous published feasibility and screening studies (19–21). Recently, several different types of smartwatches were used for cardiac rhythm analysis, including the Apple Watch, Kardia Band, Kardia Mobile and Simband (Samsung Electronics Co.) (11–14). The Apple Watch includes an irregular rhythm notification feature that detects PPG-based irregularity at rest, and can notify the user of potential AF (22). In our study, we demonstrated that the AMAZFIT^®^ could reliably distinguish an irregular pulse between AF and other rhythms, including sinus rhythm, PACs,and PVCs. The AMAZFIT^®^ for AF was found to have a sensitivity of 93.28%, a specificity of 95.35%, a PPV of 93.28%, an NPV of 95.35%, a PLR 20.06 and an NLR 0.07. Rajakariar et al. (23) reported sensitivity and specificity using the Kardia Band for identifying AF was 94.4 and 81.9% respectively, with positive and negative predictive values of 54.8 and 98.4%, respectively. These findings indicate that the AMAZFIT^®^ has excellent sensitivity and specificity for the detection of an irregular pulse from AF. In this study, we also report the sensitivity, specificity and accuracy of the AMAZFIT for PACs and PVCs. The results show that the AMAZFIT can accurately discriminate between AF, PACs, PVCs and normal ECG. McManus et al. (24) reported that a smartphone-based app showed good accuracy for PACs, at 95.5%, and for discrimination of PVCs at 96.0%. The results of our study indicate that compared with standard 12-lead ECG recordings, the AMAZFIT^®^ had excellent sensitivity and specificity for diagnosing arrhythmia such as AF, PACs and PVCs, in the older patients.

Because slower heartbeat rates can be observed in normal aging and in disease progression, bradycardia and conduction abnormalities are more commonly identified in the elderly (25). Atrioventricular block that occurs frequently or during exercise, can cause dizziness or symptoms of exertional intolerance. There have been no reports on the identification of atrioventricular block using smartwatches. In our study, we tried to explore the sensitivity and specificity of AMAZFIT^®^ for the diagnosis of first-degree atrioventricular block. The results demonstrated that AMAZFIT^®^ had a low sensitivity (32.14%) for the detection of an irregular pulse from first-degree AVB. However, the specificity of AMAZFIT^®^ for first-degree AVB (97.72%) detection was excellent. Whether AMAZFIT^®^ may serve as a useful complementary tool for first-degree AVB screening needs to be evaluated in a larger -population-based study in the future.

Unlike other smartwatches, AMAZFIT^®^ can collect single-lead ECG recordings from the chest lead in addition to limb single-lead ECG. We obtained chest lead data from all of the participants. While we collected arm lead data from only 43 (14.78%) participants, only two (4.7%) arm lead data recordings could be analyzed. Regrettably, the two recordings could not accurately reflect the heart rhythm of the patients. The advantages of AMAZFIT^®^, which collects single-lead ECG recordings of the chest lead, include convenience, small size, and long wearability. Accurate measurements with a smartwatch depend on an adequate blood flow and skin contact, and can be altered by the patient, movement of the device, environmental conditions, and ectopic beats (26). The population of this study includes adults 65 years and older. Elderly people may have loose and dry skin, which may affect the electrical conductivity of the skin, and reduce the quality of the AMAZFIT^®^ performance.

As the use of wearable devices and direct-to-consumer medical devices increases in the general population, the potential abnormalities detected by these devices are brought to the attention of clinicians. For patients diagnosed with AF, proper clinical evaluation and confirmatory ECG testing are essential. New devices such as the AMAZFIT^®^ are recommended to be integrated into patient care under physician supervision. Although important questions remain, wearable medical technology should be employed along with physician expertise.

## Conclusions

Technological limitations may prevent the widespread use of AMAZFIT^®^ arrhythmia screening in elderly patients, especially for first-degree AVB. However, AMAZFIT remains favorable compared with the conventional Holter monitoring system for symptomatic arrhythmia monitoring, despite the relatively low wearing rate in the elderly population. In the future, we look forward to more specialized arrhythmia screening tools. Cost-effective, convenient, reliable, and easy-to-apply tools for extended non-invasive AF detection would be helpful.

### Study Strenghs

This diagnostic trial revealed that detection of arrhythmia with AMAZFIT^®^ was feasible and showed a high diagnostic accuracy. The AMAZFIT^®^ smart device displayed excellent sensitivity for AF, PACs and PVCs, and a low sensitivity for first-degree AVB. In addition, we measured the HR of patients with AF using AMAZFIT^®^ and with a 12-lead ECG. The results showed that the device can accurately measure the HR of AF similarly to the 12-lead ECG.

### Study Limitations

Our study has some limitations. First, the sample size was relatively small, especially the non-AF arrhythmia cohort. Second, we only measured the cardiac rhythm in very stable patients in a motionless state. Third, all of our patients were Asian; therefore, we did not consider whether the skin color could affect the accuracy of the AMAZFIT^®^.

## Data Availability Statement

The raw data supporting the conclusions of this article will be made available by the authors, without undue reservation.

## Ethics Statement

The studies involving human participants were reviewed and approved by Institutional Review Board of West China Hospital of Sichuan University. The patients/participants provided their written informed consent to participate in this study.

## Author Contributions

JW and MY designed and supervised the study. SZ collected the data, and wrote the article. HX, YC, YL, and NZ collected the data and assisted with wrote the article. XG was responsible for the statistical design of the study and for carrying out the statistical analysis. All authors contributed to the article and approved the submitted version.

## Conflict of Interest

The authors declare that the research was conducted in the absence of any commercial or financial relationships that could be construed as a potential conflict of interest.
